# Fluctuations in waist circumference increase diabetes risk: a 4-year cohort study in 61,587 older adults

**DOI:** 10.1186/s12986-021-00627-3

**Published:** 2021-11-13

**Authors:** Linna Wu, Hongyan Liu, Zhuang Cui, Fang Hou, Xiaowen Gong, Yourui Zhang, Chunlan Lu, Hao Liu, Pei Yu

**Affiliations:** 1 NHC Key Laboratory of Hormones and Development, Tianjin Key Laboratory of Metabolic Diseases, Chu Hsien-I Memorial Hospital and Tianjin Institute of Endocrinology, Tianjin Medical University, Tianjin, 300134 China; 2 Department of Epidemiology and Health Statistics, Tianjin Medical University, Tianjin, 300010 China; 3 Community Health Service Center, Jiefang Road, Tanggu Street, Binhai New Area, Tianjin, China

**Keywords:** Fluctuations, WC, Older adults, Diabetes

## Abstract

**Purpose:**

To evaluate the effect of fluctuations in waist circumference (WC), weight, and body mass index (BMI) on the incidence of diabetes in older adults.

**Patients and methods:**

A prospective cohort of 61,587 older adults (age, 60–96 years) who did not have diabetes at study initiation was examined. Data on weight, BMI, and WC were collected, and participants were followed up until 31 December 2018. The main end point was new-onset diabetes. A Cox regression model was used to estimate the risk of diabetes (hazard ratios [HRs] and confidence intervals [CI]) in these participants.

**Results:**

During a mean follow-up of 3.6 years, being overweight (HR [95% CI] 1.87 [1.62–2.17]), obesity (1.41 [1.26–1.59]), abdominal obesity (1.42 [1.28–1.58]), and obesity plus abdominal obesity at baseline (1.93 [1.66–2.25]) increased the risk of diabetes onset. Compared with older adults who “maintained normal WC”, those who “remained abdominally obese” (HR = 1.66), “became abdominally obese” (HR = 1.58), or “achieved normal WC” (HR = 1.36) were at a higher risk of diabetes onset, as well as those with an increase in WC > 3 cm or > 5% compared with the baseline level. Weight gain or loss > 6 kg or weight gain > 5%, increase or decrease in BMI > 2 kg/m^2^, or an increase in BMI > 10% were associated with a higher diabetes risk. The diabetes risk was reduced by 19% in overweight older adults who exercised daily.

**Conclusion:**

For older adults, WC, BMI, and healthy weight maintenance reduce the diabetes risk. The findings may provide evidence for developing guidelines of proper weight and WC control for older adults.

## Introduction

Diabetes is a chronic non-communicable disease, and its prevalence is rapidly increasing globally. Diabetes causes various health issues and contributes to a huge economic burden due to its serious long-term complications. The prevalence of diabetes in Chinese adults is 12.8%, suggesting that diabetes is a serious public health concern in China [1].

The prevalence of overweight and obesity in Chinese adults tripled, from 11.7 to 29.2%, between 1991 and 2009 [2]. Overweight, obesity, and abdominal obesity are strong risk factors for diabetes in all age groups. In a 23-year-long prospective study of British men, increased body mass index (BMI) accounted for 26% of the increased incidence of type 2 diabetes (T2DM), suggesting a strong link between obesity and diabetes risk [3]. Moreover, it has been shown that weight gain is an independent risk factor for diabetes regardless of baseline BMI levels, and the risk positively correlates with the extent of weight gain. Thus, weight is also an important factor influencing the development of diabetes.

Little is known as to how the fluctuations in waist circumference (WC) and body weight changes affect the risk of diabetes in older adults. In older adults, a decline in organ function and changes in body composition can cause serious complications, resulting in higher morbidity and mortality. The risk of diabetes in older adults may therefore differ from that in young and middle-aged populations. China, with its increasingly ageing population, has a diabetes prevalence of 20.4% among older adults [4]. Thus, a better understanding of the association between diabetes incidence and WC, weight, BMI, and the changes in these parameters in older adults is necessary to develop better individualised weight management strategies for older adults.

In this study, we aimed to examine the effect of WC, weight, BMI, and the changes in these parameters, as well as the effect of physical activity on diabetes incidence in older individuals.

## Materials and methods

### Study population and examinations

Participants in our study were recruited between January and December 2015 from older adults aged 60 years and above living in Tianjin Binhai New Area. This area is a new industrialised coastal special zone located in north-eastern China; it has relatively good economic conditions and is urbanised. The participants were local residents from 37 different neighbourhood committees who underwent annual physical examinations (funded by the Government of Binhai, New Area; free of charge for local residents aged 60 years or older) at nearby community health service centres. Physical examinations included the following anthropometric measurements: weight, height, WC, systolic blood pressure (SBP), and diastolic blood pressure (DBP). Laboratory tests evaluated white blood cell (WBC) and platelet (PLT) counts, as well as levels of haemoglobin (Hgb), total cholesterol (TC), triglyceride (TG), fasting blood glucose (FBG), alanine transaminase (ALT), aspartate transaminase (AST), total bilirubin (TBIL), serum creatinine (Scr), and blood urea nitrogen (BUN). A face-to-face interview was conducted to collect information on sociodemographic characteristics. All physical examination data were recorded in a unified online database.

In total, 162,826 people underwent physical examination in 2015, and our study included 79,389 of them without severe cardiovascular disease, cerebrovascular disease, hepatic or renal dysfunction, and malignant tumours. We excluded participants with diabetes and those who had self-reported using hypoglycaemic drugs before the study (n = 17,625). Therefore, 61,764 individuals were included and followed up until they were diagnosed with diabetes or until the end of 2018. During the follow-up period, 177 participants who did not undergo annual physical examinations were also excluded. Finally, 61,587 participants were included in the final cohort analysis (median follow-up time: 3.6 years, range: 0.3–4.0 years). Informed consent was obtained from all the participants, and the study protocol was approved by the Institutional Review Board of Tianjin Medical University Metabolic Diseases Hospital.

### The definition of BMI and WC

Body weight, height, and BMI were measured in accordance with the standard protocol. WC was measured as the horizontal girth of the waist through the midpoint between the anterior superior iliac spine and the lower margin of the 12th rib. According to baseline BMI, individuals were categorised as underweight (< 18.5 kg/m^2^), normal weight (18.5–23.9 kg/m^2^), overweight (24–27.9 kg/m^2^), and obese (≥ 28 kg/m^2^). According to baseline WC, individuals were categorised as having normal WC (< 85 cm in women or < 90 cm in men) or abdominal obesity (≥ 85 cm in women or ≥ 90 cm in men). The cut-offs were based on the Guidelines for the Prevention and Control of Overweight and Obesity in Chinese Adults. Based on changes in BMI from baseline to the final examination, individuals were classified into four groups: “remained underweight or maintained normal weight” (BMI maintained at < 24 kg/m^2^), “became obese or overweight” (BMI changed from < 24 to ≥ 24 kg/m^2^), “became normal or underweight” (BMI changed from ≥ 24 to < 24 kg/m^2^), and “remained obese or overweight” (BMI maintained at ≥ 24 kg/m^2^). Based on changes in WC status, participants were classified as “retained normal WC”, “became abdominally obese”, “achieved normal WC”, and “remained abdominally obese”. The variations in weight, BMI, and WC during the follow-up were defined as the differences between the values at baseline in 2015 and at diabetes diagnosis or at the final examination in 2018. The percentage changes in weight, BMI, and WC during the follow-up were defined as the variations divided by the corresponding baseline value × 100. We combined the underweight and normal weight groups in certain analyses because the number of individuals with newly diagnosed diabetes in these groups was low.

### The definition of diabetes

Glucose levels were measured using fasting serum samples (blood samples collected after overnight fasting for at least 8 h) obtained during the annual physical examinations and using separate FBG tests performed every 3–6 months. A standard 75-g glucose tolerance test was also administered if the preliminary FBG level was higher than 6.1 mmol/L to further examine the 2-h postprandial plasma glucose value. The presence of diabetes was determined in accordance with the criteria formulated by the World Health Organization (WHO) expert committee on diabetes in 1999.

### Other covariates

Data on age, sex, smoking habits (never smoker, past smoker, or current smoker), alcohol consumption (never, less than once a week, more than once a week, or every day), dietary preference (vegetarian diet, animal-based diet, or balanced diet), frequency of physical activity (never, less than once a week, more than once a week, or every day), and medication history (use of hypoglycaemic agents, anti-hypertensive drugs, statins, and psychotropic drugs) were collected from the initial face-to-face interview. Blood pressure was measured twice on the right arm after at least 5 min of resting, and the average of the two values was recorded. The presence of hypertension was determined based on an SBP ≥ 140 mmHg and/or DBP ≥ 90 mmHg, or the use of anti-hypertensive drugs.

### Statistical analysis

Continuous variables were described as mean values ± standard deviation (mean ± SD) and categorical variables as numbers and percentages (%). Participants were categorised according to baseline BMI and WC levels; changes in BMI and WC status; variations in weight, BMI, and WC; and the frequency of physical activity. Sex- (male, female) and age-specific (age < 75 years; age ≥ 75 years) subgroup analyses were also performed. Time at risk was calculated as the interval in days from the date of the initial examination in 2015 to the date of diabetes diagnosis or the date of the final examination in 2018. Cox proportional hazards regression models were used to estimate the hazard ratios (HRs) and 95% confidence intervals (95% CIs) for new-onset diabetes, using values in individuals with normal BMI and WC; those with stable weight, BMI, and WC; and those who did not exercise regularly as the reference. The regression models were adjusted for confounders, including baseline age, ALT and TBIL levels, alcohol consumption, and statin use. All statistical analyses were performed using SPSS version 22.0. All *P* values were based on two-sided tests, and *P* < 0.05 was considered statistically significant.

## Results

During a median follow-up of 3.6 years, among the 61,587 older individuals without diabetes at baseline, we identified 1414 new-onset cases of diabetes (6.4/1000 person-years); the annual incidence of diabetes was 0.76%.

### Characteristics of patients with newly diagnosed diabetes

Compared with older individuals without diabetes, those diagnosed with diabetes were younger; had a higher prevalence of hypertension; used statins more frequently; had a higher baseline BMI, WC, and TG, FBG, ALT, and AST levels; had lower TBIL levels; and had a lower frequency of smoking and daily drinking (*P* < 0.05) (Table1).

**Table 1:** Characteristics of patients with newly diagnosed diabetes, and hazard ratios (HRs) for diabetes in older individuals (2015–2018) calculated using univariate and multivariate analysis

	New-onset diabetes		Univariate analysis		Multivariate analysis
	No	Yes	HR (95% CI)	*P* value	HR (95% CI)
Number	60,173 (97.7%)	1414 (2.3%)			
Sex					
Men (%)	26,573 (97.7%)	638 (2.3%)	1.00	0.428	
Women (%)	33,600 (97.7%)	776 (2.3%)	0.96 (0.86–1.06)		
Age (years)	67.68 ± 6.09	66.98 ± 5.90	0.98 (0.97–0.99)	< 0.001	
60–70	40,415 (97.6%)	1013 (2.4%)	1.00	0.002	1.00*
71–80	16,749 (98.0%)	344 (2.0%)	0.83 (0.73–0.93)		0.82 (0.72–0.92)
> 80	3009 (98.1%)	57 (1.9%)	0.76 (0.58–1.00)		0.75 0.58–0.99)
Hypertension					
No (%)	26,811 (97.9%)	577 (2.1%)	1.00	0.008	
Yes (%)	33,362 (97.6%)	837 (2.4%)	1.15 (1.04–1.28)		
SBP (mmHg)	127.47 ± 13.51	127.44 ± 12.56	1.00 (0.99–1.00)	0.641	
< 120	16,261 (97.8%)	359 (2.2%)	1.00	0.016	
120–139	36,026 (97.6%)	896 (2.4%)	1.11 (0.98–1.25)		
≥ 140	7886 (98.0%)	159 (2.0%)	0.88 (0.73–1.06)		
DBP (mmHg)	78.71 ± 7.63	78.51 ± 7.05	1.00 (0.99–1.00)	0.202	
< 80	28,102 (97.7%)	667 (2.3%)	1.00	0.026	
80–89	26,782 (97.6%)	649 (2.4%)	1.01 (0.91–1.12)		
≥ 90	5289 (98.2%)	98 (1.8%)	0.76 (0.61–0.94)		
BMI (kg/m^2^)	24.66 ± 3.08	25.56 ± 3.19	1.09 (1.07–1.11)	< 0.001	
< 18.5	811 (99.1%)	7 (0.9%)	0.48 (0.23–1.00)	< 0.001	0.48 (0.23–1.02)
18.5–23.9	26,577 (98.2%)	479 (1.8%)	1.00		1.00*
24–27.9	24,622 (97.5%)	641 (2.5%)	1.43 (1.27–1.61)		1.28 (1.14–1.45)
≥ 28	8163 (96.6%)	287 (3.4%)	1.91 (1.65–2.21)		1.48 (1.28–1.72)
WC (cm)	84.94 ± 8.54	87.01 ± 9.15	1.03 (1.02–1.03)	< 0.001	
< 85 (Women)/ < 90 (Men)	37,389 (98.0%)	748 (2.0%)	1.00	< 0.001	
≥ 85 (Women)/ ≥ 90 (Men)	22,784 (97.2%)	666 (2.8%)	1.44 (1.30–1.60)		
TC (mmol/L)	5.24 ± 1.12	5.24 ± 1.32	0.99 (0.95–1.04)	0.993	
< 5.2	30,436 (97.6%)	746 (2.4%)	1.00	0.083	
≥ 5.2	29,737 (97.8%)	668 (2.2%)	0.91 (0.82–1.01)		
TG (mmol/L)	1.54 ± 0.95	1.72 ± 1.07	1.12 (1.09–1.16)	< 0.001	
< 1.7	43,392 (97.9%)	929 (2.1%)	1.00	< 0.001	
≥ 1.7	16,781 (97.2%)	485 (2.8%)	1.33 (1.19–1.48)		
FBG (mmol/L)	5.35 ± 0.91	5.96 ± 1.35	1.08 (1.07–1.09)	< 0.001	
< 6.1	52,939 (98.3%)	911 (1.7%)	1.00	< 0.001	1.00*
6.1–6.9	5819 (95.2%)	294 (4.8%)	2.82 (2.47–3.22)		2.75 (2.40–3.14)
≥ 7.0	1415 (87.1%)	209 (12.9%)	7.85 (6.75–9.13)		7.34 (6.29–8.56)
ALT (U/L)	21.72 ± 14.67	24.74 ± 14.71	1.01 (1.00–1.01)	< 0.001	
< 40	56,788 (97.8%)	1266 (2.2%)	1.00	< 0.001	1.00*
≥ 40	3385 (95.8%)	148 (4.2%)	1.90 (1.60–2.25)		1.42 (1.20–1.70)
AST (U/L)	23.62 ± 12.17	24.67 ± 11.11	1.00 (1.00–1.01)	0.003	
< 45	58,603 (97.8%)	1348 (2.2%)	1.00	< 0.001	
≥ 45	1570 (96.0%)	66 (4.0%)	1.76 (1.38–2.26)		
TBIL (µmol/L)	13.91 ± 6.02	13.49 ± 5.74	0.99 (0.98–1.00)	0.007	
< 20	53,499 (97.7%)	1286 (2.3%)	1.00	0.008	1.00*
≥ 20	6674 (98.1%)	128 (1.9%)	0.78 (0.65–0.94)		0.70 (0.58–0.84)
Scr (µmol/L)	75.60 ± 22.05	75.89 ± 19.40	1.00 (1.00–1.01)	0.705	
< 106	56,698 (97.7%)	1332 (2.3%)	1.00	0.976	
≥ 106	3475 (97.7%)	82 (2.3%)	1.00 (0.80–1.25)		
BUN (mmol/L)	5.66 ± 2.00	5.59 ± 1.89	0.98 (0.96–1.01)	0.236	
< 7.1	51,425 (97.6%)	1243 (2.4%)	1.00	0.007	
≥ 7.1	8748 (98.1%)	171 (1.9%)	0.80 (0.68–0.94)		
Hgb (g/L)	138.76 ± 15.11	139.32 ± 16.04	1.00 (1.00–1.01)	0.268	
< 110 (Women)/ < 120 (Men)	1663 (97.7%)	39 (2.3%)	1.02 (0.74–1.41)	0.888	
≥ 110 (Women)/≥120 (Men)	58,510 (97.7%)	1375 (2.3%)	1.00		
WBC (*10^12^/L)	6.02 ± 3.96	6.21 ± 3.56	1.01 (1.00–1.02)	0.073	
< 4.0	3464 (98.7%)	45 (1.3%)	0.54 (0.40–0.72)	< 0.001	
4.0–9.9	55,856 (97.6%)	1349 (2.4%)	1.00		
≥ 10	853 (97.7%)	20 (2.3%)	0.98 (0.63–1.53)		
PLT (*10^12^/L)	209.57 ± 55.85	209.24 ± 53.14	1.00 (1.00–1.00)	0.747	
< 100	56,174 (97.7%)	1328 (2.3%)	1.55 (1.06–2.27)	0.007	
100–299	726 (96.4%)	27 (3.6%)	1.00		
≥ 300	3273 (98.2%)	59 (1.8%)	0.76 (0.58–0.98)		
Smoking status					
Never	45,412 (75.5%)	1132 (80.1%)	1.00	< 0.001	
Past	2816 (4.7%)	58 (4.1%)	0.82 (0.63–1.06)		
Current	11,945 (19.9%)	224 (15.8%)	0.74 (0.64–0.85)		
Alcohol consumption					
Never	50,475 (83.9%)	1242 (87.8%)	1.00	0.001	1.00*
Less than once a week	4363 (7.3%)	77 (5.4%)	0.71 (0.57–0.90)		0.67 (0.53–0.84)
More than once a week	1507 (2.5%)	30 (2.1%)	0.80 (0.56–1.15)		0.70 (0.49–1.01)
Everyday	3828 (6.4%)	65 (4.6%)	0.69 (0.54–0.88)		0.66 (0.51–0.85)
Diet					
Animal-based	732 (1.2%)	16 (1.1%)	1.00	0.828	
Balanced	57,227 (95.1%)	1349 (95.4%)	1.08 (0.66–1.78)		
Vegetarian diet	2214 (3.7%)	49 (3.5%)	1.00 (0.57–1.76)		
Physical activity					
Never	12,706 (21.1%)	307 (21.7%)	1.00	0.676	
Less than once a Week	3193 (5.3%)	82 (5.8%)	1.06 (0.83–1.35)		
More than once a week	13,467 (22.4%)	304 (21.5%)	0.93 (0.79–1.09)		
Everyday	30,807 (51.2%)	721 (51.0%)	0.98 (0.85–1.12)		
History of drug use					
Statins					
No (%)	59,856 (97.7%)	1401 (2.3%)	1.00	0.046	1.00**
Yes (%)	317 (96.1%)	13 (3.9%)	1.74 (1.01–3.01)		1.85 (1.07–3.20)
Psychotropics					
No (%)	60,018 (97.7%)	1410 (2.3%)	1.00	0.752	
Yes (%)	155 (97.5%)	4 (2.5%)	1.17 (0.44–3.13)		

BMI, body mass index; WC, waist circumference; SBP, systolic blood pressure; DBP, diastolic blood pressure; WBC, white blood cells; Hgb, haemoglobin; PLT, platelet; TC, total cholesterol; TG, triglyceride; FBG, fasting blood glucose; ALT, alanine transaminase; AST, aspartate transaminase; TBIL, total bilirubin; Scr, serum creatinine; BUN, blood urea nitrogen

*P < 0.01, **P < 0.05

In the univariate regression analysis, overweight, obesity, abdominal obesity, hyperlipemia, use of statins, impaired glucose regulation, or abnormal liver function were significantly associated with diabetes onset. Individuals with higher levels of TBIL and those with habit of smoking and drinking more frequently had a lower diabetes risk. The multivariate regression analysis showed that the increase in BMI, FBG and ALT levels, and the use of statins were independent risk factors for diabetes (Table 1). The risk of diabetes in overweight or obese individuals was 1.28- and 1.48-times higher, respectively, than that in individuals with normal weight at baseline. Compared with older individuals with normal FBG levels at baseline, those with FBG levels of 6.1–7.0 mmol/L or higher than 7.0 mmol/L had a 1.75- and 6.34-times higher risk of diabetes, respectively. Compared with older individuals with ALT levels less than 40 U/L, those with ALT levels of 40 U/L and higher had a 42% higher risk of diabetes. Older individuals using statins had an 85% higher risk of diabetes than those not using statins.

### Baseline BMI and WC levels are associated with diabetes

When the complete study population was examined, baseline BMI and WC levels were associated with the incidence of diabetes. Obese older men with normal WC had the highest risk of diabetes (Table 2), followed by obese older men with abdominal obesity, overweight older men with abdominal obesity, and older men with normal weight and WC. Similarly, obese older women with abdominal obesity had the highest risk of diabetes, followed by overweight older women with abdominal obesity and older women with normal weight and WC. Among older adults aged less than 75 years, obese individuals with abdominal obesity had the highest risk of diabetes, followed by obese individuals with normal WC and overweight individuals with abdominal obesity. Among older adults aged 75 years and older, overweight individuals with normal WC had the highest risk of diabetes, followed by overweight individuals with abdominal obesity.

**Table 2:** Hazard ratios (HRs) for diabetes according to baseline body mass index (BMI) and waist circumference (WC) values in older individuals (2015–2018)

BMI (kg/m^2^)	Normal WC			Abdominal obesity		
	< 24	24–27.9	≥ 28	< 24	24–27.9	≥ 28
Number	24,806	12,556	775	3068	12,707	7675
New cases of diabetes (%)	433 (1.7%)	293 (2.3%)	22 (2.8%)	53 (1.7%)	348 (2.7%)	265 (3.5%)
No. per 1000 person-years	4.9	6.6	8.0	4.9	7.7	9.7
HR (95% CI)*^△^	1.00	1.33 (1.15–1.54)	1.60 (1.05–2.46)	0.99 (0.74–1.31)	1.53 (1.33–1.77)	1.93 (1.66–2.25)
Men						
Number	11,184	6405	366	1047	5145	3064
New cases of diabetes (%)	204 (1.8%)	16 (2.5%)	15 (4.1%)	14 (1.3%)	144 (2.8%)	99 (3.2%)
No. per 1000 person-years	5.2	7.1	11.5	3.8	8.1	9.0
HR (95% CI)*^△^	1.00	1.38 (1.13–1.70)	2.24 (1.32–3.78)	0.75 (0.44–1.29)	1.54 (1.24–1.91)	1.76 (1.38–2.24)
Women						
Number	13,622	6151	409	2021	7562	4611
New cases of diabetes (%)	229 (1.7%)	131 (2.1%)	7 (1.7%)	39 (1.9%)	204 (2.7%)	166 (3.6%)
No. per 1000 person-years	4.7	5.8	4.8	5.4	7.5	10.1
HR (95% CI)*^△^	1.00	1.24 (1.00–1.54)	0.99 (0.47–2.10)	1.16 (0.82–1.62)	1.57 (1.30–1.90)	2.10 (1.72–2.56)
Age < 75 years						
Number	20,390	10,866	673	2448	10,827	6758
New cases of diabetes (%)	377 (1.8%)	253 (2.3%)	21 (3.1%)	44 (1.8%)	310 (2.9%)	247 (3.7%)
No. per 1000 person-years	5.2	6.5	8.8	5.1	8.1	10.2
HR (95% CI)*^△^	1.00	1.26 (1.08–1.48)	1.68 (1.08–2.61)	0.96 (0.71–1.32)	1.52 (1.31–1.77)	1.95 (1.66–2.29)
Age ≥ 75 years						
Number	4416	1690	102	620	1880	917
New cases of diabetes (%)	56 (1.3%)	40 (2.4%)	1 (1.0%)	9 (1.5%)	38 (2.0%)	18 (2.0%)
No. per 1000 person-years	3.6	6.7	2.7	4.1	5.7	5.5
HR (95% CI)**^△^	1.00	1.92 (1.28–2.89)	0.80 (0.11–5.77)	1.13 (0.56–2.28)	1.60 (1.06–2.41)	1.57 (0.92–2.67)

*P < 0.001, **P < 0.05, CI, confidence interval

^△^The HRs were adjusted for confounders including baseline age, ALT and TBIL levels, alcohol consumption, and statin use

### The change of BMI or WC status increases the incidence of diabetes

Compared with older adults who “remained underweight or maintained normal weight”, those who “became overweight or obese” (HR = 1.96), “remained overweight or obese” (HR = 1.88), or “became underweight or achieved normal weight” (HR = 1.72) had a significantly higher risk of diabetes onset. Compared with older adults who “remained normal WC”, those who “remained abdominally obese” (HR = 1.66), “became abdominally obese” (HR = 1.58), or “achieved normal WC” (HR = 1.36) were also at a significantly higher risk of diabetes onset (Table 3).

**Table 3:** Hazard ratios (HRs) for diabetes according to body mass index (BMI) and waist circumference (WC) status in older individuals (2015–2018)

	Remained normal/underweight	Normal/underweight to overweight/obese	Overweight/obese to normal/underweight	Remained overweight/obese
Number	22,133 (35.9%)	5741 (9.3%)	4110 (6.7%)	29,603 (48.1%)
New cases of diabetes (%)	322 (1.5%)	164 (2.9%)	103 (2.5%)	825 (2.8%)
No. per 1000 person-years	4.1	8.1	7.5	7.8
HR (95% CI)*^△^	1.00	1.96 (1.62–2.37)	1.72 (1.38–2.15)	1.88 (1.65–2.14)

	Retained normal waist circumference	Normal waist circumference to abdominal obesity	Abdominal obesity to normal waist circumference	Remained abdominally obese
Number	30,030 (48.8%)	8107 (13.2%)	5086 (8.2%)	18,382 (29.8%)
No. new cases of diabetes (%)	524 (1.7%)	224 (2.8%)	121 (2.4%)	545 (3.0%)
No. per 1000 person-years	4.9	7.8	6.7	8.4
HR (95% CI)^*△^	1.00	1.58 (1.36–1.85)	1.36 (1.11–1.65)	1.66 (1.48–1.88)

*P < 0.001, CI, confidence interval

^△^The HRs were adjusted for confounders including baseline age, ALT and TBIL levels, alcohol consumption, and statin use

### The change of weight, BMI, or WC increases the incidence of diabetes

During the follow-up period, weight remained unchanged in 24.9% of the older adults; however, 38.1% of them lost weight and 37.0% gained weight. The risk of diabetes was significantly higher in those who gained or lost > 6 kg of weight, or showed a 5–10% or higher increase in weight relative to baseline than in those with unchanged weight. BMI remained unchanged, increased, or decreased during the follow-up in 20.2%, 36.5%, and 43.3% of the older adults, respectively. The risk of diabetes was significantly higher in individuals with a BMI increase or decrease of > 2 kg/m^2^ or a BMI increase of > 10% relative to baseline than in those with unchanged BMI (Table 4).

**Table 4:** Hazard ratios (HRs) for diabetes according to weight, body mass index (BMI), and waist circumference (WC) variation in older individuals (2015–2018)

Weight change (kg)	< − 6	− 6 to − 3	− 3 to 0	0	0 to 3	3 to 6	> 6
Number New-onset diabetes (%)	3694 122 (3.3%)	6062	13,730	15,313	13,365	5417	4006
		134 (2.2%)	283 (2.1%)	348 (2.3%)	239 (1.8%)	129 (2.4%)	159 (4.0%)
New-onset diabetes/1000 person-years	9.7	6.2	5.8	6.5	5.0	6.7	11.4
HR (95% CI)*^△^	1.48 (1.21–1.83)	0.98 (0.81–1.20)	0.91 (0.77–1.06)	1.00	0.79 (0.67–0.93)	1.05 (0.86–1.29)	1.77 (1.47–2.14)

Weight change (%)	< − 10%	− 10% to − 5%	− 5% to 0	0	0 to 5%	5% to 10%	> 10%
Number	3103	5348	10,054	19,206	10,100	6796	6980
New-onset diabetes (%)	67 (2.2%)	130 (2.4%)	233 (2.3%)	393 (2.0%)	217 (2.1%)	167 (2.5%)	207 (3.0%)
New-onset diabetes/1000 person-years	6.1	6.8	6.5	5.8	6.0	6.9	8.4
HR (95% CI)*^△^	1.08 (0.83–1.40)	1.19 (0.97–1.45)	1.14 (0.97–1.34)	1.00	1.06 (0.90–1.25)	1.21 (1.01–1.45)	1.48 (1.25–1.75)

BMI change (kg/m^2^)	< − 2	− 2 to − 1	− 1 to 0	0	0 to 1	1 to 2	> 2	
Number	4655	6613	11,206	12,422	11,869	7453	7369	
New-onset diabetes (%)	142 (3.1%)	145 (2.2%)	226 (2.0%)	288 (2.3%)	228 (1.9%)	139 (1.9%)	246 (3.3%)	
New-onset diabetes/1000 person-years	8.6	6.1	5.6	6.5	5.4	5.2	9.5	
HR (95% CI)*^△^	1.32 (1.08–1.62)	0.95 (0.78–1.16)	0.87 (0.73–1.04)	1.00	0.83 (0.70–0.99)	0.80 (0.65–0.98)	1.45 (1.22–1.72)	

BMI change (%)	< − 10%	− 10% to − 5%	− 5% to 0	0	0 to 5%	5% to 10%	> 10%
Number	2917	6073	13,543	12,422	13,782	7045	5805
New-onset diabetes (%)	84 (2.9%)	150 (2.5%)	281 (2.1%)	288 (2.3%)	258 (1.9%)	156 (2.2%)	197 (3.4%)
New-onset diabetes/1000 person-years	8.1	6.9	5.8	6.5	5.2	6.2	9.7
HR (95% CI)*^△^	1.27 (0.99–1.62)	1.07 (0.88–1.30)	0.90 (0.76–1.06)	1.00	0.81 (0.68–0.95)	0.96 (0.79–1.16)	1.48 (1.23–1.77)

WC change (cm)	< − 6	− 6 to -3	− 3 to 0	0	0 to 3	3 to 6	> 6
Number	5451	5027	8027	19,206	8242	6272	9362
New-onset diabetes (%)	129 (2.4%)	121 (2.4%)	180 (2.2%)	393 (2.0%)	167 (2.0%)	159 (2.5%)	265 (2.8%)
New-onset diabetes/1000 person-years	6.6	6.7	6.4	5.8	5.7	7.1	8.0
HR (95% CI)*^△^	1.17 (0.96–1.43)	1.17 (0.96–1.44)	1.10 (0.92–1.31)	1.00	1.00 (0.83–1.19)	1.25 (1.04–1.50)	1.41 (1.20–1.65)

WC change (%)	< − 10%	− 10% to − 5%	− 5% to 0	0	0 to 5%	5% to 10%	> 10%
Number	3103	5348	10,054	19,206	10,100	6796	6980
New-onset diabetes (%)	67 (2.2%)	130 (2.4%)	233 (2.3%)	393 (2.0%)	217 (2.1%)	167 (2.5%)	207 (3.0%)
New-onset diabetes/1000 person-years	6.1	6.8	6.6	5.8	6.0	6.9	8.4
HR (95% CI)*^△^	1.08 (0.83–1.40)	1.19 (0.97–1.45)	1.14 (0.97–1.34)	1.00	1.06 (0.90–1.25)	1.21 (1.01–1.45)	1.48 (1.25–1.75)

*P < 0.01, CI, confidence interval

^△^The HRs were adjusted for confounders including baseline age, ALT and TBIL levels, alcohol consumption, and statin use

During the follow-up period, the WC remained unchanged in 37.2%, increased in 30.0%, and decreased in 38.3% of the older adults. The risk of diabetes was significantly higher among older adults who showed a WC increase of 3–6 cm or more and among those with WC increase of 5–10% or more relative to baseline than among those with unchanged WC (Table 4).

### Daily exercise reduces the risk of diabetes, especially for overweight old individuals

Our final analysis involved the assessment of new-onset diabetes risk according to the frequency of physical activity at different baseline BMI levels (Table 5). We found that a higher frequency of physical activity was associated with a lower risk of new-onset diabetes. Interestingly, the risk of diabetes in overweight older adults who exercised daily was 19% lower than that in normal-BMI individuals who did not exercise regularly.

**Table 5:** Hazard ratios (HRs) for diabetes according to baseline body mass index (BMI) and frequency of physical exercise in older individuals (2015–2018)

	Never	Less than once a week	More than once a week	Everyday
Number				
BMI < 24 kg/m^2^	6315	1477	6279	13,803
24 kg/m^2^ ≤ BMI < 28 kg/m^2^	4972	1352	5569	13,370
BMI ≥ 28 kg/m^2^	1726	446	1923	4355
No. of new cases of diabetes (%)				
BMI < 24 kg/m^2^	105 (1.7%)	24 (1.6%)	101 (1.6%)	256 (1.9%)
24 kg/m^2^ ≤ BMI < 28 kg/m^2^	146 (2.9%)	42 (3.1%)	141 (2.5%)	312 (2.3%)
BMI ≥ 28 kg/m^2^	56 (3.2%)	16 (3.6%)	62 (3.2%)	153 (3.5%)
No. per 1000 person-years				
BMI < 24 kg/m^2^	4.7	3.8	4.6	5.2
24 kg/m^2^ ≤ BMI < 28 kg/m^2^	8.2	8.7	7.1	6.6
BMI ≥ 28 kg/m^2^	9.1	10.1	9.0	9.9
HR (95% CI)^△^				
BMI < 24 kg/m^2^	1.00	0.98 (0.63–1.53)	0.98 (0.74–1.29)	1.16 (0.92–1.45)
24 kg/m^2^ ≤ BMI < 28 kg/m^2^	1.00	1.08 (0.77–1.52)	0.88 (0.69–1.11)	0.81 (0.67–0.99)
BMI ≥ 28 kg/m^2^	1.00	0.76 (0.48–1.21)	0.48 (0.18–1.28)	0.67 (0.38–1.16)

*CI* confidence interval

^△^The HRs were adjusted for confounders including baseline age, ALT and TBIL levels, alcohol consumption, and statin use

## Discussion

Our study demonstrated that obesity, overweight, and abdominal obesity at baseline and the increase or decrease in body weight, BMI, and WC during follow-up were associated with an increased risk of diabetes in older adults. The risk of new-onset diabetes in obese older adults with abdominal obesity was higher in women than in men. Daily exercise was able to reduce the risk of diabetes by 19% for overweight older adults.

Our study suggested that metabolic disorders, including obesity and high blood glucose and serum lipid levels, can significantly increase the risk of diabetes. A cohort study involving 51,405 Korean men [5] showed that individuals who developed T2DM were likely to have a higher BMI, FBG, and serum lipid levels, which is consistent with our findings. Similar conclusions have been drawn in the meta-analysis by Lotta et al. [6] who showed that compared with healthy individuals, metabolically unhealthy individuals had a higher risk of T2DM, irrespective of the BMI category.

In the univariate regression analysis in our study, individuals with the habit of smoking and drinking more frequently had a lower diabetes risk. We assume that it was because individuals without the habits of smoking and drinking gave up smoking and drinking after experiencing multiple chronic diseases, and they were also more susceptible to diabetes in the future.

Our study showed that older adults with higher BMI at baseline had a higher risk of developing diabetes. Overweight at baseline was a strong predictor of diabetes risk, independent of weight gain. Previous studies have shown that the relative risk (RR) of diabetes in individuals with BMI 30.0–34.9 kg/m^2^ or ≥ 35 kg/m^2^ was up to 20.1- or 38.8-times higher than that among those with BMI < 23 kg/m^2^ [7]. In a retrospective cohort study of 1257 parous women [8], initial BMI and BMI after 28–48 years of follow-up were found to be strongly associated with the diabetes risk, which increased with weight gain.

In our study, among overweight or obese older adults, the risk of diabetes was higher in abdominally obese individuals than in those with normal WC, and obese older adults with abdominal obesity had the highest risk. The WC mainly reflects visceral fat content. The increase in body fat percentage is associated with a higher risk of diabetes, even in those with normal weight or underweight [9]. The National Diabetes and Metabolic Disease Survey [10] showed that compared with individuals with normal weight and WC, individuals with obesity, and the combination of the two had 1.88-, 1.12-, and 2.19-times higher odds of diabetes, respectively. This could be because an increase in WC results in the release of more free fatty acids from adipose tissue, causing lipid toxicity in beta cells, which in turn leads to a further decline of the islet cell secretory function.

Our study suggested that the increase in WC (> 3 cm or > 5%) was also associated with an increased risk of diabetes, so that the increase in WC may be a predictor of T2DM. The American Health Professionals Study [11] showed that 20% of the risk of diabetes could be attributed to an increase of > 2.5 cm in WC. The diabetes risk in individuals with a WC increase of 14.6 cm or more increased by 70% during a 4-year follow-up period.

In addition, our study showed that the risk of new-onset diabetes in obese older adults with abdominal obesity was higher in women than in men. A previous investigation has shown that the proportion of older adults with central obesity is higher among women, and that the risk of diabetes is higher among adults with central obesity than among those with low BMI and WC [10]. Women generally have a higher percentage of fat, including total fat mass, subcutaneous thigh fat, and subcutaneous abdominal fat, and they are more sensitive to the negative effects of excess fat accumulation. Increasing levels of visceral fat were associated with an approximately three-fold increase in diabetes risk in women, while the risk in men increased modestly by 20% [12]. The central distribution of adipose tissue has a greater influence on the incidence of non-insulin-dependent diabetes in women than in men and may contribute to an increased risk of diabetes [13]. Together, these findings suggest that abdominal obesity may be a stronger risk factor for diabetes among women.

In our age-dependent subgroup analysis, the RR of diabetes in obese or abdominally obese adults aged 75 years and more was lower than that in individuals aged less than 75 years. According to a cross-sectional survey of Tianjin residents in 2017, the incidence of diabetes is the highest in individuals aged 75 years (0.75%), and then rapidly declines with age (0.4% in individuals aged 85 years). However, in our study, this trend only appeared in obese or abdominally obese older adults. The effect of overweight and obesity on the incidence of diabetes gradually weakens with increasing age. Furthermore, older adults may be less easily affected by a slight increase in the risk due to weight gain than younger individuals. Considering the close connection between obesity and several life-threatening conditions, such as cardiovascular disease and stroke [14], older obese individuals may be more likely to have a combination of these diseases and thus experience early death. The survivors may not be as susceptible to such disorders, including diabetes, which could explain the low incidence of diabetes in adults aged over 75 years.

Our study showed that the risk of diabetes was higher among those who became obese, remained obese, or achieved a normal BMI than among those who remained non-obese. Weight gain and large weight fluctuations were also independent risk factors of diabetes, regardless of baseline BMI levels [15], and the risk positively correlated with the extent of weight gain [16, 17]. More than 9% of diabetes cases could be avoided if obese individuals became non-obese [18]. Some studies on weight loss through lifestyle interventions, such as the Da Qing study from China and the Diabetes Prevention Program, have reported a 43% and 34% decrease in diabetes risk, respectively, in individuals with prediabetes after long-term follow-up [19, 20].

When we analysed how the variation in weight and BMI and a high increase or decrease in weight or BMI affects the risk of new-onset diabetes, a U-shaped curve was found. Older adults who became obese or overweight had the highest risk of diabetes, followed by those who remained overweight or obese and those who achieved normal weight or became underweight; the corresponding risk of diabetes also increased in older adults with high weight loss (> 6 kg during the follow-up). A possible reason is that the included individuals in our study were older adults. Namely, during weight loss, older adults may lose proportionately more muscle mass than younger individuals. This loss of skeletal muscle and the increased proportion of fat may contribute to increased insulin resistance, which may weaken the benefit derived from the weight loss. Weight change across adulthood increases all-cause and cause-specific mortality [21].

Our study showed that daily exercise was able to reduce the risk of diabetes, especially for overweight old adults. Specifically, daily exercise reduced the risk of diabetes by 19% for overweight older adults. Physical activity is important for diabetes prevention. Physical activity can effectively reduce the risk of diabetes [22–25], and an appropriate increase in energy consumption can effectively regulate postprandial insulin secretion and improve the glucose metabolism status [26]. This effect can persist for up to 10 years. However, the target population that can gain most benefits from regular exercise is yet to be identified. The effect of moderate- or high-intensity exercise on diabetes prevention is more obvious in obese individuals [27, 28]. Individuals with normal weight can benefit more from exercise or gain the same benefits as obese people and those with prediabetes [29]. In our study, daily exercise reduced the risk of diabetes by 19% for overweight older adults, but the risk of diabetes did not show a significant decline for individuals with normal weight who exercised daily. This may be because individuals with normal body weight already had a low risk of diabetes. Moreover, the body fat content in individuals with normal weight was lower; thus, a further significant decline in body weight and fat would be challenging.

Our study has some strengths. It is the first large-scale prospective cohort study on the effect of obesity indicators and their changes on the incidence of diabetes in older adults from urban areas of northern China. Our study included over 60,000 individuals, and the data were maintained in an updated database, which ensured the accuracy and integrity of such large amount of data. Moreover, subgroup analyses were performed according to sex, age, and baseline BMI levels, and confounders were adjusted for to minimise their influence. Furthermore, the participants enrolled in our study were community residents, and not inpatients, and our cohort was thus well representative of the general older adult population.

Nevertheless, our study had several limitations. First, the duration of the follow-up was only 4 years, and the long-time effects of the changes in weight, BMI, and WC did not appear; therefore, we plan to prolong the follow-up time in further research. Second, our participants were from a coastal city in northern China; hence, our data may not be generalisable to individuals of other ethnicities and those living in other areas.

## Conclusion

Our study demonstrated that obesity, overweight, and abdominal obesity at baseline and the increase or decrease in body weight, BMI, and WC during follow-up are associated with an increased risk of diabetes in older adults. Daily exercise can reduce the risk of diabetes, and it has a greater benefit in overweight older adults. The findings may provide evidence for developing guidelines of proper weight control for older adults.

## Data Availability

The datasets used and/or analyzed during the current study are available from the corresponding author on reasonable request.

## Abbreviations

BMI: Body mass index
T2DM: Type 2 diabetes
WC: Waist circumference
SBP: Systolic blood pressure
DBP: Diastolic blood pressure
WBC: White blood cell
PLT: Platelet
Hgb: Haemoglobin
TC: Total cholesterol
TG: Triglyceride
FBG: Fasting blood glucose
ALT: Alanine transaminase
AST: Aspartate transaminase
TBIL: Total bilirubin
Scr: Serum creatinine
BUN: Blood urea nitrogen
WHO: World Health Organization
HRs: Hazard ratios
95% CIs: 95% Confidence intervals
RR: Relative risk
